# A study on the temporal patterns, anatomical distribution, and influencing factors of radiation therapy-associated second primary cancers in gynecological malignancies

**DOI:** 10.3389/fonc.2026.1726727

**Published:** 2026-01-29

**Authors:** Yuling Ye, Xiuhua Li, Ting Lin, Xian Lin, Hongqian Ke, Hongqing Wu, Yang Sun

**Affiliations:** Department of Gynecology, Fujian Cancer Hospital, Clinical Oncology School of Fujian Medical University, Fuzhou, China

**Keywords:** clinical characteristics analysis, gynecological malignancies, influencing factors, radiotherapy, secondary primary cancers

## Abstract

**Objective:**

To systematically analyze the clinical characteristics of post-radiotherapy secondary primary cancers (SPC) after treatment for gynecological malignancies, with the aim of informing and optimizing tumor management strategies.

**Methods:**

A retrospective collection of clinical data for 36 patients with gynecological malignancies who underwent radiotherapy at our hospital from January 2004 to December 2021.

**Results:**

The first primary cancer was predominantly cervical cancer (31 cases, 86.1%), with endometrial cancer accounting for 5 cases. Radiotherapy-related variables included the median radiotherapy duration of 42.5 days, external irradiation dose of 49.375 Gy, and total dose of 69.5 Gy; FIGO stages I/II/III were 7/18/11; lymph node metastasis in 6 cases; HPV positive in 20 cases; surgical history in 18 cases and chemotherapy history in 30 cases. The SPC cohort had a median onset age of 58 years and a median interval of 8 years after radiotherapy, with primary sites mostly in the rectum/colon and uterus; 24 cases occurred outside the radiotherapy field. Surgical, radiotherapy, and chemotherapy histories all influenced the timing and site of SPC. The median onset age after radiotherapy and interval to SPC were significantly longer in the cervical cancer group than in the endometrial cancer group (P = 0.01), and most SPC occurred within 5–10 years.

**Conclusions:**

The pathological features and treatment history of the initial cancer were associated with the timing and location of SPC. Analyses of post-radiotherapy SPC characteristics provide a scientific basis for optimizing clinical management and reducing SPC risk.

## 1 Introduction

Gynecological malignancies remain a significant threat to women’s health worldwide. Epidemiological data indicate a rising incidence of these tumors, profoundly impacting patient survival, quality of life, and healthcare systems (1, 2). While advancements in multimodal treatment—including surgery, radiotherapy, chemotherapy, targeted therapy, and immunotherapy—have considerably improved survival rates and reduced disease-related mortality (3–5). In particular, the role of radiotherapy in improving local control rates and prolonging overall survival has become increasingly prominent. Nevertheless, the long-term side effects associated with the treatment have gradually emerged, and the potential long-term adverse events have gradually become the focus of attention (6). Among these, radiotherapy-associated secondary primary cancer (SPC) represents a clinically significant long-term complication whose mechanisms, temporal patterns, clinical behavior, and impact on survival remain incompletely understood (7). SPC is defined as a new, independently arising malignancy in patients with a history of radiotherapy, distinct from the primary tumor in anatomical site, histology, and molecular characteristics, and not attributable to metastasis or recurrence (8, 9). As cancer survival rates improve, radiotherapy-associated SPC has emerged as a critical challenge in long-term oncologic care.

In recent years, with the continuous advancement of radiotherapy technology, radiotherapy has become an important component of the treatment plan for at least 50% of cancer patients, and is also associated with the occurrence of multiple-site SPC (10). Increasing evidence suggests that radiotherapy may increase SPC risk through multiple pathobiological pathways, including radiation-induced genetic damage, inflammatory microenvironment alterations, synergistic effects with chemotherapeutic agents, and prolonged exposure in secondary irradiation areas, etc. Large-scale international cohort studies and retrospective analyses consistently demonstrate significantly higher SPC incidence among radiotherapy-treated patients compared to non-irradiated counterparts with similar primary tumors (11). For instance, Hou et al. estimated that approximately 6.6% of second solid tumors may be attributable to radiotherapy for primary cancers, indicating that SPC is not a negligible concern in long-term follow-up and survivorship care (12). Similarly, Zhu et al.’s SEER database analysis revealed a substantially elevated absolute excess risk of SPC (107.99 per 10,000 person-years) in young patients with head and neck squamous cell carcinoma receiving postoperative radiotherapy (13). These studies suggest that there is an association between radiotherapy and SPC, but the specific incidence rate, clinical manifestations, and prognostic characteristics still require further systematic and targeted research to clarify.

Despite these findings, the clinical presentation and incidence patterns of SPC following radiotherapy for gynecological malignancies remain inadequately characterized. Therefore, this study aims to retrospectively analyze clinical data from gynecological cancer patients who developed SPC after radiotherapy, with the goal of elucidating clinical characteristics and potential risk factors to provide an evidence basis for optimizing tumor management strategies and improving follow-up plans.

## 2 Materials and methods

### 2.1 Patients

This study retrospectively collected a total of 36 patients with SPC after radiotherapy for gynecological malignant tumors who were admitted to Fujian Cancer Hospital from January 2004 to December 2021. Inclusion criteria: Inclusion criteria were as follows: (1) Pathologically confirmed cervical cancer and endometrial cancer; (2) Having complete clinical data; (3)Received radiotherapy. Exclusion criteria were as follows: (1) Had other malignant tumors at the initial diagnosis; (2) Had severe functional disorders of important organs such as the heart, liver, and kidneys; (3) Incomplete clinical data. The tumor stage of cervical cancer patients was determined according to the 2018 International Federation of Gynecology and Obstetrics (FIGO) staging criteria; the 2009 FIGO staging criteria for endometrial cancer patients was used for the tumor stage of endometrial cancer patients. The tumor stage of all patients was determined by at least two experienced clinicians through a comprehensive analysis of all examination results. Finally, 36 patients met the inclusion criteria. This study was conducted in accordance with the principles of the Helsinki Declaration and was approved by the Ethics Committee of Fujian Cancer Hospital (K2025-329-01).

### 2.2 Data collection

The retrospective collection of data on the pathological type, tumor stage, presence or absence of lymph node metastasis, HPV infection status, age at radiotherapy, start and end times of radiotherapy, external irradiation dose of radiotherapy, total radiotherapy dose, presence or absence of surgery, and presence or absence of chemotherapy for the first cancer from the hospital’s electronic medical record system; the collection of data on the age of onset of SPC, the interval time after radiotherapy to the onset, the location, located within the radiotherapy field or outside the field, whether to undergo surgery, whether to receive chemotherapy and radiotherapy. Radiotherapy techniques included two-dimensional radiotherapy (2D-RT, n=11), three-dimensional conformal radiotherapy (3D-CRT, n=11), intensity-modulated radiotherapy (IMRT, n=10), and volumetric modulated arc therapy (VMAT, n=5). The external beam prescription dose was 45–50.4 Gy delivered in 23–28 fractions. In addition, 26 patients received brachytherapy at a dose of 6 Gy per session over 2–5 sessions. 2D-RT typically employed two- or four-field opposed arrangements. IMRT plans usually utilized 6–9 coplanar or non-coplanar beams, which were individually optimized based on the patient’s specific anatomy. During treatment planning, organs at risk (OARs) were routinely contoured and evaluated, including the spinal cord, femoral heads, bladder, rectum, sigmoid colon, small bowel, and ovaries.

The radiation field was defined based on reconstructed isodose distributions from original treatment plans, primarily referencing the clinical target volume (CTV) boundary. SPCs were classified as ‘inside the field’ if their geometric center was within this boundary, and ‘outside the field’ if entirely beyond it. For lesions adjacent to the field edge, a distance of >1 cm from the boundary was used as the cutoff for ‘outside field’ classification.

### 2.3 Follow-up

Post-treatment follow-up is typically recommended according to a structured schedule: baseline by a follow-up at 1 month after completion of therapy; then every 3 months during years 1-2; every 6 months during years 3-5; and annually thereafter. During these follow-ups, evaluations commonly include physical examination, laboratory tests (biochemistry, complete blood count, and tumor markers), and imaging studies (e.g., chest CT and abdominal MRI).

### 2.4 Statistical analyses

This study is a descriptive case series and does not include a control group; thus, inferences about causality or risk cannot be made. Data analysis was conducted using SPSS 26.0 statistical software. Measurement data that followed a normal distribution were expressed as mean ± standard deviation, while data that did not follow a normal distribution were expressed as median. Comparisons between groups were performed using Student’s t-test or Mann-Whitney U test; count data were compared using chi-square test or Fisher’s exact test. All analyses were two-tailed, and p-values < 0.05 were considered statistically significant.

## 3 Results

### 3.1 Clinical characteristics

This study presents a descriptive analysis of a cohort of 36 patients. It is important to note that due to the overall limited sample size and the particularly small endometrial cancer subgroup (n=5), all subsequent between-group comparisons and subgroup analyses should be regarded as exploratory. As shown in **Table 1**, the main pathological types were cervical cancer (n = 31, 86.1%) and endometrial cancer (n = 5, 13.9%). The median age at initial radiotherapy was 50 years. Initial cancer FIGO stages included stage I (n = 7), stage II (n = 18), and stage III (n = 11). Initial cancer with lymph node metastasis was present in 6 cases. HPV infection was positive in 20 cases. The median days of the first radiotherapy was 42.5 days. The median external radiation radiotherapy dose was 49.375 Gy, and the median total dose was 69.5 Gy. Surgery was performed in 18 cases, and chemotherapy was administered in 30 cases. As shown in **Table 2**, the median age of onset for SPC was 58 years; the median interval from onset was 8 years; the main SPC location were the colon and the uterus; 24 patients had their tumors outside the radiotherapy field; 19 patients underwent surgery; 11 patients received radiotherapy; and 23 patients received chemotherapy.

**Table 1:** Clinical characteristics of 36 patients with initial cancer.

Characteristics	Number (n, %)
Pathological pattern	
Cervical cancer	31 (86.11%)
Endometrial cancer	5 (13.89%)
Age at initial radiotherapy (year)	
<50	17 (47.22%)
≥50	19 (52.78%)
Initial cancer FIGO stage	
Stage I	7 (19.44%)
Stage II	18(50.00%)
Stage III	11 (30.56%)
Initial cancer with lymph node metastasis	
Yes	6 (16.67%)
No	30 (83.33%)
HPV infection	
Positive	20 (55.56%)
Negative	16 (44.44%)
Days of the first radiotherapy	42.5 (35.75, 63.5)
External radiation radiotherapy dose	49.375 (47.7, 50)
Total dose	69.5 (49.99, 81.75)
Radiotherapy techniques	
2D-RT	11 (30.56%)
3D-CRT	11 (30.56%)
IMRT	10 (27.78%)
VMAT,	5 (13.89%)
Brachytherapy	
Yes	26 (72.22%)
No	10 (27.78%)
Initial cancer surgery	
Yes	18 (50.00%)
No	18 (50.00%)
Initial cancer chemotherapy	
Yes	30 (83.33%)
No	6 (16.67%)

**Table 2:** Clinical characteristics of 36 patients with SPC.

Characteristics	Number (n, %)
Age onset of SPC (years)	
<58	14 (38.89%)
≥58	22 (61.11%)
Interval from onset (years)	
<8	17 (47.22%)
≥8	19 (52.78%)
SPC location	
Vagina	3 (8.33%)
Uterus	11 (30.56%)
Colorectum	13 (36.11%)
Vulva	4 (11.11%)
Ovary	2 (5.56%)
Bladder	1 (2.78%)
Right ilium	1 (2.78%)
Right thigh	1 (2.78%)
Radiotherapy irradiation field	
Within	12 (33.33%)
Outside	24 (66.67%)
Surgery	
Yes	19 (52.78%)
No	17 (47.22%)
Radiotherapy	
Yes	11 (30.56%)
No	25 (69.44%)
Chemotherapy	
Yes	23 (63.89%)
No	13 (36.11%)

### 3.2 Analysis of clinical characteristics of cervical cancer and endometrial cancer

This study conducted a comparative analysis of the differences in clinical variables and radiotherapy-related factors between cervical cancer (n=31) and endometrial cancer (n=5) (**Table 3**). The results indicated that among the variables examined, only the days of the first radiotherapy, whether initial cancer surgery, and interval from onset (years) (P < 0.05). The other variables, including tumor stage, age at initial radiotherapy, radiotherapy dose, total dose, lymph node metastasis, etc., did not present statistically significant differences. This exploratory finding suggests a potential association between the timing of radiotherapy initiation, surgical history, and the interval to SPC onset across the two pathological types, warranting investigation in larger studies. These observed associations may reflect differences in treatment contexts and early disease management between the groups.

**Table 3:** Analysis of clinical characteristics of cervical cancer and endometrial cancer.

Characteristics	Cervical cancer	Endometrial cancer	Chi-Squared test	
n, %	31, %	5, %	χ²	P value
Initial cancer FIGO stage				
Stage I	5 (13.89%)	2 (5.86%)	1.590	0.558
Stage II	16 (44.44%)	2 (5.86%)		
Stage III	10 (27.78%)	1 (2.78%)		
Age at initial radiotherapy (years)				
<50	16 (44.44%)	1 (2.78%)	1.726†	0.342
≥50	15 (41.67%)	4 (11.11%)		
Days of the first radiotherapy				
<42.5	11 (30.56%)	5 (13.89%)	7.056†	0.012
≥42.5	20 (55.56%)	0 (0.00%)		
External radiation radiotherapy dose				
<49.375	16 (44.44%)	2 (5.86%)	0.226†	0.999
≥49.375	15 (41.67%)	3 (8.33%)		
Total dose				
<69.50	14 (38.89%)	4 (11.11%)	2.032†	0.338
≥69.50	17 (47.22%)	1 (2.78%)		
Initial cancer chemotherapy				
Yes	26 (72.22%)	4 (11.11%)	0.045†	0.999
No	5 (13.89%)	1 (2.78%)		
Initial cancer surgery				
Yes	13 (36.11%)	5 (13.89%)	5.645†	0.045
No	18 (50.00%)	0 (0.00%)		
Initial cancer with lymph node metastasis				
Yes	5 (13.89%)	1 (2.78%)	0.045†	0.999
No	26 (72.22%)	4 (11.11%)		
HPV infection				
Positive	17 (47.22%)	3 (8.33%)	0.045†	0.999
Negative	14 (38.89%)	2 (5.56%)		
Age of onset of SPC (years)				
<58	11 (36.11%)	3 (8.33%)	1.060†	0.357
≥58	20 (55.56%)	2 (5.56%)		
Interval from onset (years)				
<8	12 (33.33%)	5 (13.59%)	6.309†	0.016
≥8	19 (52.48%)	0 (0.00%)		
SPC location				
Reproductive organs	18 (50.00%)	2 (2.78%)	0.566†	0.451
Non-reproductive organs	13 (36.11%)	3 (11.11%)		
Radiotherapy irradiation field				
Within	11 (30.56%)	1 (2.78%)	0.452†	0.646
Outside	20 (55.56%)	4 (11.11%)		
SPC surgery				
Yes	16 (44.44%)	3 (8.33%)	0.118†	0.999
No	15 (41.67%)	2 (5.56%)		
SPC radiotherapy				
Yes	10 (27.78%)	1 (2.78%)	0.296†	0.664
No	21 (58.33%)	4 (11.11%)		
SPC chemotherapy				
Yes	21 (58.33%)	2 (5.56%)	1.396†	0.328
No	10 (27.78%)	3 (8.33%)		

†indicates the use of Fisher’s exact test.

### 3.3 Analysis of the age at onset of initial cancer and SPC

**Figure 1:**
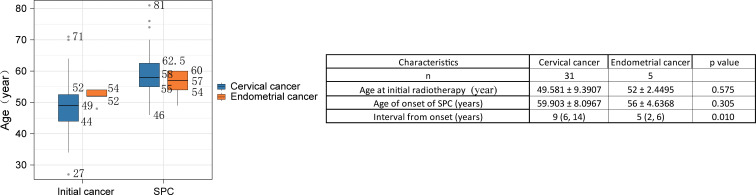
Comparison of the age distribution of initial cancer and SPC.

As shown in **Figure 1**, among the primary cancers, the median age of onset for patients with cervical cancer was 49 years (range: 27–71 years), while that for patients with endometrial cancer was 52 years (range: 49–60 years). In the SPC, the median age of onset for patients with cervical cancer was 58 years (range: 46–81 years), and for patients with endometrial cancer, it was 57 years (range: 48–54 years). No significant differences were observed between the two groups of patients in terms of the age of radiotherapy for primary cervical cancer and endometrial cancer, as well as the age of onset of their respective SPC. Additionally, among patients with primary cervical cancer, the median interval from the initial cancer to the development of SPC after radiotherapy was 9 years (range: 2–24 years); in contrast, for patients with primary endometrial cancer, the median interval was 5 years (range: 1–6 years). The difference in the time from radiotherapy to the onset of SPC between the two groups of patients was statistically significant (p = 0.01). Although a statistically significant difference was observed in the median interval from radiotherapy to SPC onset between the cervical and endometrial cancer groups (9 *vs*. 5 years, p=0.01), the very small size of the endometrial cancer subgroup (n=5) markedly limits the reliability and generalizability of this comparison. This finding should therefore be viewed primarily as a preliminary signal for future research.

**Figure 2:**
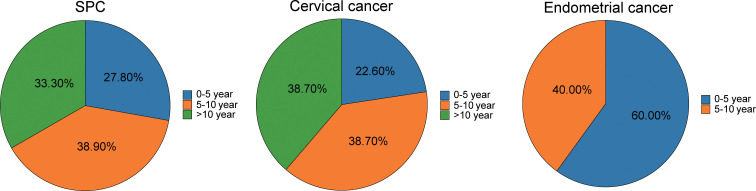
The proportion of time intervals between the onset of SPC, cervical cancer and endometrial cancer in 36 cases.

As illustrated in **Figure 2**, among the 36 patients who developed an SPC following radiotherapy, the timing of SPC occurrence distributed as follows: 10 cases (27.8%) occurred within ≤5 years after the initial cancer diagnosis, 14 cases (38.9%) occurred between 5–10 years, and 12 cases (33.3%) arose after >10 years. Of the 31 patients with cervical cancer as their initial malignancy, SPC developed in 7 patients (22.6%) within ≤5 years, 12 patients (38.7%) during the 5–10 year interval, and 12 patients (38.7%) beyond 10 years. Among the 5 patients with endometrial cancer, SPC occurred in 3 patients (60%) within ≤5 years and in 2 patients (40%) between 5–10 years; no SPC cases were observed beyond 10 years in this subgroup. These findings indicate that SPC following cervical cancer most commonly arises 5–10 years after initial diagnosis, whereas SPC in patients with endometrial cancer generally occurs earlier, within the first 5 years.

**Figure 3:**
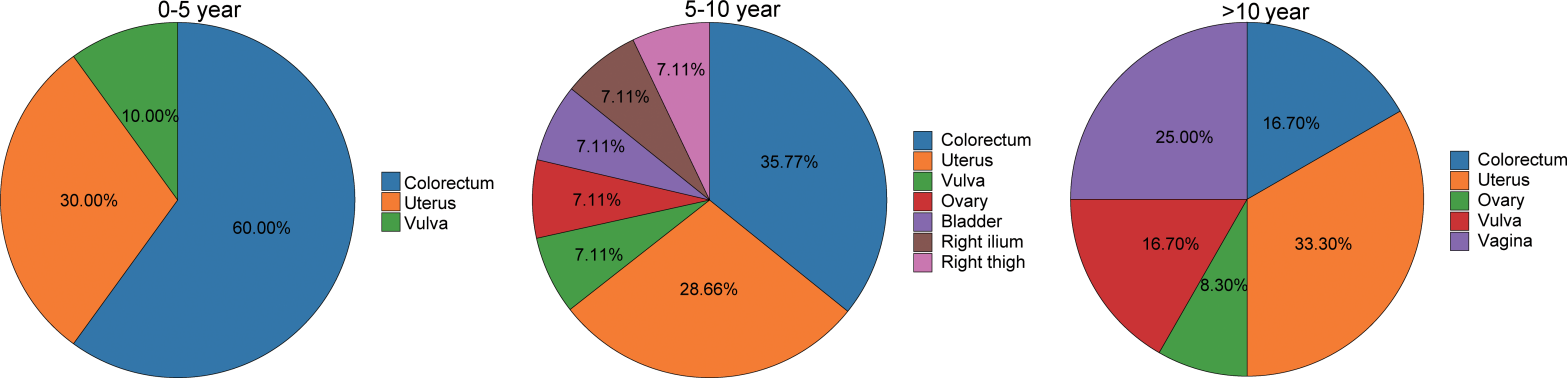
The distribution of the occurrence sites of SPC at different time intervals of onset.

As depicted in **Figure 3**, the anatomical distribution of SPC sites varied across different time periods after radiotherapy. Within the first 5 years, SPCs predominantly occurred in the colorectum and uterus. Between 5–10 years, the colorectum and uterus remained the predominant sites. After more than 10 years, the most common sites were the uterus, colorectum, and vulva. In summary, the colorectum and uterus are the primary sites where SPC is most likely to develop in patients with gynecological malignancies following radiotherapy.

### 3.4 Analyze the clinical characteristics of the initial cancer and its relationship with SPC

**Figure 4:**
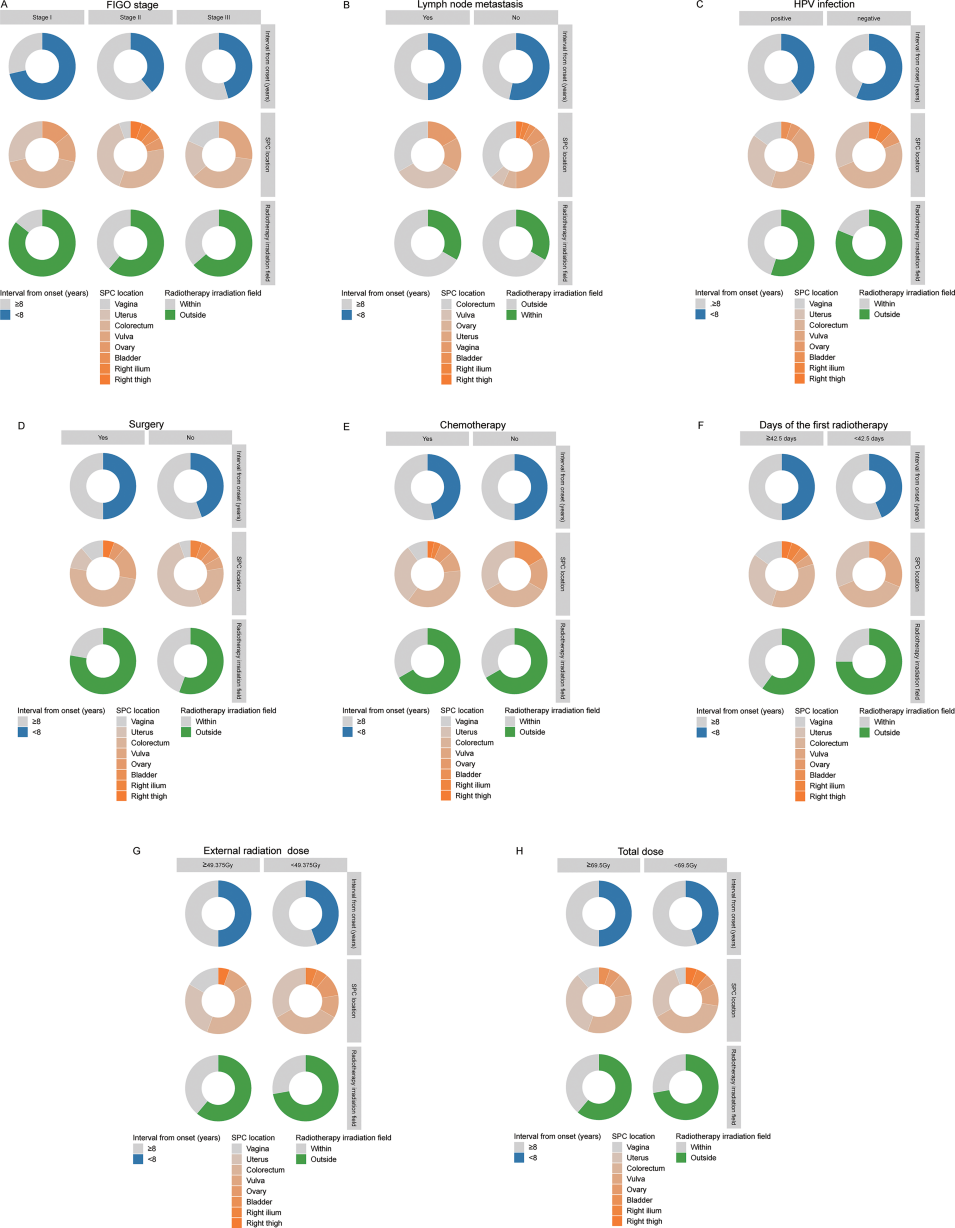
Correlation analysis of the clinical characteristics of the first-stage cancer and SPC. **(A)** FIGO installment; **(B)** Lymph node metastasis; **(C)** HPV infection; **(D)** Surgery; **(E)** Chemotherapy; **(F)** Days of the first radiotherapy; **(G)** External radiation radiotherapy dose; **(H)** Total dose.

**Figure 4A** illustrates the distribution of SPC timing and location stratified by initial FIGO stage. Among patients with stage I, SPCs were predominantly observed within 8 years after radiotherapy completion. In contrast, for stages II and III, SPC more commonly occurred beyond the 8-years post-treatment period. Across all stage groups (I, II, and III), the colorectum and uterus were the most frequent SPC sites, with a majority of cases located outside the original radiation field. **Figure 4B** depicts the pattern among patients without lymph node metastasis, showing that SPC development most frequently occurred more than 8 years post-radiotherapy. The primary SPC sites in this subgroup were also the colon/rectum and uterus, with a high proportion situated outside the irradiated volume. **Figure 4C** presents data for HPV-positive patients, indicating a tendency for SPCs to arise after 8 years. The major involved sites included the colorectum, uterus, and vulva, with most instances occurring outside the previous radiation field. **Figure 4D** demonstrates the profile for patients with a history of surgery. In this subgroup, the timing of SPC onset did not show a clear association with the interval from radiotherapy completion. The predominant SPC locations were the colon/rectum and vulva, again with a high frequency of outside-field occurrences. **Figure 4E** outlines the characteristics for patients who received chemotherapy, among whom SPCs mostly developed after 8 years. The main sites were the colorectum and uterus, accompanied by frequent outside-field recurrences. **Figures 4F-H** summarize the findings for patients stratified by radiotherapy duration (≥42.5 days) and total dose (≥49.375 Gy and ≥69.5 Gy, respectively). Within these parameter-based subgroups, the timing of SPC onset did not display a clear differential pattern. The principal SPC sites remained the colorectum, uterus, and vagina, with the majority of cases located outside the irradiation field.

## 4 Discussion

Radiotherapy-related SPC represent a significant long-term adverse outcome following radiation treatment. The peak incidence typically occurs 5 to 15 years or later after radiotherapy, reflecting the cumulative nature of risk, which is associated with initial treatment intensity and radiation dose (14, 15). The overall incidence of SPC varies considerably across tumor types and increases with age, particularly in middle-aged and elderly populations. Gender differences are evident, as SPC spectra differ for certain primary tumors, suggesting influences from reproductive organ exposure, hormonal milieu, and sex-specific genetic and environmental factors (16, 17). Geographic and population variations also exist, attributable to disparities in radiotherapy access, diagnostic resources, lifestyle factors, and genetic background (18). Furthermore, radiotherapy-related parameters and combined treatments collectively influence SPC risk (11, 12). A comprehensive understanding of SPC epidemiology and clinical features aids in identifying high-risk populations, optimizing follow-up and screening, and guiding risk-benefit assessments in radiotherapy planning.

This study examined the clinical characteristics and patterns of SPC following radiotherapy for after radiotherapy, addressing questions on timing, location, and potential influences of primary cancer stage and treatment history. The primary cancer was predominantly cervical cancer with high HPV prevalence. SPC occurred most frequently in the colorectum and uterus, often outside the radiation field, indicating involvement of non-radiation factors. Although the median age at SPC onset was relatively late, significant differences were observed between primary cancer groups in time to SPC development, with a longer interval in cervical cancer patients. These findings highlight that: 1) SPC timing relates closely to primary tumor biology and treatment history; 2) the post-radiotherapy pelvic region remains a key site for SPC, underscoring the need for long-term surveillance of irradiated and adjacent areas; and 3) differences in SPC patterns across primary cancer subtypes support individualized follow-up strategies. As a descriptive case series with a limited sample size, the primary aim of this study was to characterize patterns and temporal distributions of SPCs following radiotherapy for gynecological cancers, not to establish definitive causal relationships or risk factors. Consequently, all subgroup findings reported here should be considered hypothesis-generating, highlighting important avenues for future, more statistically robust research. The research results provide evidence for optimizing radiotherapy target area design, formulating individualized long-term follow-up plans, and early detection of SPC, while also revealing the diversity and complexity of post-radiotherapy SPC.

A key finding of our descriptive analysis is that a majority of SPCs (24/36) developed outside the originally irradiated volume. This observation suggests that a substantial proportion of these second malignancies in our cohort may represent sporadic second primary cancers rather than being directly induced by radiation exposure. This aligns with the understanding that the risk of radiation-induced malignancy is generally highest within or at the margin of the treatment field, where doses are highest, while the risk decreases substantially with distance and lower scatter doses (19). The high rate of out-of-field SPCs underscores the multifactorial etiology of second cancers in cancer survivors, which may include genetic predisposition, shared environmental risk factors, or late effects of systemic therapies such as chemotherapy. Therefore, our findings highlight the importance of comprehensive, long-term surveillance for all second cancers in this population, not only those within the prior radiation field.

The development of SPC following radiotherapy demonstrates complex associations with multiple clinical variables. Our descriptive analysis revealed a significant difference in the median time to SPC onset between cervical cancer and endometrial cancer cohorts. This observation suggests that different initial cancer treatment plans may be related to the timing and location of SPC through factors such as radiotherapy dose, duration, surgery, and chemotherapy. Our findings further indicate that SPC events frequently occur during extended follow-up periods, highlighting the clinical importance of sustained long-term monitoring. Particular attention to previously irradiated areas during follow-up examinations may facilitate earlier detection of potential SPCs. These observations are consistent with broader oncological literature demonstrating that post-radiotherapy SPC patterns vary substantially across tumor types, radiation techniques, and patient-specific factors. Gonzalez et al. reported differential SPC rates between breast cancer (5-6%) and endometrial cancer (11%) populations, suggesting tumor-type-specific considerations in radiotherapy-related SPC risk (11). Liang et al. observed that postoperative radiotherapy significantly elevated SPC risk in triple-negative breast cancer patients with BRCA germline mutations, though no significant interaction was identified between BRCA1/2 mutations and radiotherapy effects (20). On the other hand, the research team from Greece evaluated the bladder and rectal carcinogenic risks of VMAT and 3D-CRT in cervical cancer, and found that VMAT slightly increased the risk of bladder cancer but slightly decreased the risk of rectal cancer, and both were significantly higher than those in the non-radiotherapy group (21). Journy et al. found that during radiotherapy for breast cancer, if the dose and irradiation area of the esophagus increase, the risk of esophageal cancer after radiotherapy will rise (22). The occurrence of a notable proportion of SPCs outside the primary radiation field, as observed in our cohort, underscores the multifactorial etiology of these second malignancies and necessitates consideration of alternative or synergistic explanatory mechanisms. First, the median age at SPC diagnosis in our cohort (58 years) aligns with the typical peak incidence age for several sporadic carcinomas, suggesting that some out-of-field SPCs may represent age-dependent, *de novo* malignancies independent of prior radiation. Furthermore, chemotherapy exposure in a significant subset of patients may contribute, as certain agents carry independent carcinogenic risks not confined to radiation fields. In addition, underlying genetic predispositions could confer a generalized, lifetime increase in the risk of multiple primary cancers, irrespective of prior radiotherapy fields. Moreover, shared lifestyle or environmental factors, such as the high prevalence of HPV infection in this population, may serve as common oncogenic drivers for malignancies at disparate sites. Finally, the context of detection must be considered; the intensive surveillance inherent to long-term follow-up after cancer treatment inherently increases the probability of detecting new malignancies anywhere in the body, which may influence the observed distribution of SPCs. These studies collectively suggest that the risk of SPC after radiotherapy is not only related to the type of primary tumor, but also to the selection of radiotherapy technology, dose distribution, and the patient’s genetic background. Therefore, clinical practice should comprehensively assess the necessity of radiotherapy, optimize the design of radiotherapy target areas and dose distribution, and strengthen long-term follow-up and multidisciplinary intervention to reduce the risk of SPC and improve the early diagnosis rate. A holistic risk assessment strategy that integrates these multifaceted factors is essential for personalized patient management.

Carrying out research on the clinical characteristics of SPC after radiotherapy for gynecological malignant tumors has multiple clinical values: clarifying the clinical manifestations and occurrence patterns of SPC, improving the sensitivity of early diagnosis and follow-up; systematically summarizing the occurrence characteristics of SPC, helping to formulate individualized risk assessment and management measures, including the optimization of radiotherapy dose and range, and prevention strategies for high-risk groups; by analyzing the influencing factors of radiotherapy, providing scientific basis for the clinical optimization of radiotherapy plans, reducing the risk of SPC occurrence, alleviating the disease burden, and promoting the innovation and development of related treatment technologies, ultimately improving the survival level and quality of life of patients with gynecological malignant tumors. Therefore, systematically exploring the characteristics of SPC after radiotherapy for gynecological malignant tumors from both clinical and research perspectives has significant theoretical and practical application values. However, the interpretation of our results must be tempered by recognition of key methodological limitations. The small overall sample size, and particularly the minimal number of endometrial cancer cases (n=5), significantly reduces the statistical power of comparisons and increases the uncertainty around our estimates, including those that reached conventional significance levels. Additionally, as this is a retrospective study, detailed dose-volume histogram parameters (e.g., Dmax, Dmean, V20) for all organs at risk (OARs) were not fully archived for some early cases, which may limit a more granular analysis of dose-response relationships. Therefore, our findings are primarily descriptive and hypothesis-generating. Future multi-center collaborative studies, analyses of larger cancer registry databases, or prospective case-control/cohort studies with complete dosimetric data are essential to validate the observed patterns, more reliably investigate risk factors in rare subgroups, and assess causality.

## 5 Conclusion

The pathological features and treatment history of the initial cancer can affect the timing and location of SPC. Analyses of post-radiotherapy SPC characteristics provide a scientific basis for optimizing clinical management and reducing SPC risk.

## Data Availability

The raw data supporting the conclusions of this article will be made available by the authors, without undue reservation.

## References

[1] Siegel RL Giaquinto AN Jemal A . Cancer statistics, 2024. CA Cancer J Clin. (2024) 74:12–49. doi: 10.3322/caac.21820, PMID: 38230766

[2] Mayadev JS Ke G Mahantshetty U Pereira MD Tarnawski R Toita T . Global challenges of radiotherapy for the treatment of locally advanced cervical cancer. Int J Gynecol Cancer. (2022) 32:436–45. doi: 10.1136/ijgc-2021-003001, PMID: 35256434 PMC8921593

[3] Crosbie EJ Kitson SJ McAlpine JN Mukhopadhyay A Powell ME Singh N . Endometrial cancer. Lancet. (2022) 399:1412–28. doi: 10.1016/S0140-6736(22)00323-3, PMID: 35397864

[4] Harkenrider MM Abu-Rustum N Albuquerque K Bradfield L Bradley K Dolinar E . Radiation therapy for endometrial cancer: an american society for radiation oncology clinical practice guideline. Pract Radiat Oncol. (2023) 13:41–65. doi: 10.1016/j.prro.2022.09.002, PMID: 36280107

[5] Cohen PA Jhingran A Oaknin A Denny L . Cervical cancer. Lancet. (2019) 393:169–82. doi: 10.1016/S0140-6736(18)32470-X, PMID: 30638582

[6] Journy N Mansouri I Allodji RS Demoor-Goldschmidt C Ghazi D Haddy N . Volume effects of radiotherapy on the risk of second primary cancers: A systematic review of clinical and epidemiological studies. Radiother Oncol. (2019) 131:150–9. doi: 10.1016/j.radonc.2018.09.017, PMID: 30316563

[7] Zeng M Lin A Jiang A Qiu Z Zhang H Chen S . Decoding the mechanisms behind second primary cancers. J Transl Med. (2025) 23:115. doi: 10.1186/s12967-025-06151-9, PMID: 39856672 PMC11762917

[8] Ye Y Li X Lin T Lin X Ke H Wu H . Sarcoma arising in irradiated bone; report of 11 cases. Cancer. (1948) 1:3–29. doi: 10.1002/1097-0142(194805)1:1<3::AID-CNCR2820010103>3.0.CO;2-7, PMID: 18867438

[9] Sale KA Wallace DI Girod DA Tsue TT . Radiation-induced Malignancy of the head and neck. Otolaryngol Head Neck Surg. (2004) 131:643–5. doi: 10.1016/j.otohns.2004.05.012, PMID: 15523441

[10] Hymel R Jones GC Simone CB 2nd . Whole pelvic intensity-modulated radiotherapy for gynecological Malignancies: A review of the literature. Crit Rev Oncol Hematol. (2015) 94:371–9. doi: 10.1016/j.critrevonc.2014.12.015, PMID: 25600840 PMC4420646

[11] Berrington de Gonzalez A Curtis RE Kry SF Gilbert E Lamart S Berg CD . Proportion of second cancers attributable to radiotherapy treatment in adults: a cohort study in the US SEER cancer registries. Lancet Oncol. (2011) 12:353–60. doi: 10.1016/S1470-2045(11)70061-4, PMID: 21454129 PMC3086738

[12] Hou N Wang Z Ling Y Hou G Zhang B Zhang X . Radiotherapy and increased risk of second primary cancers in breast cancer survivors: An epidemiological and large cohort study. Breast. (2024) 78:103824. doi: 10.1016/j.breast.2024.103824, PMID: 39442313 PMC11532779

[13] Zhu X Zhou J Zhou L Zhang M Gao C Tao L . Association between postoperative radiotherapy for young-onset head and neck cancer and long-term risk of second primary Malignancy: a population-based study. J Transl Med. (2022) 20:405. doi: 10.1186/s12967-022-03544-y, PMID: 36064552 PMC9446763

[14] Chow JCH Tam AHP Cheung KM Lee VHF Chiang CL Tong M . Second primary cancer after intensity-modulated radiotherapy for nasopharyngeal carcinoma: A territory-wide study by HKNPCSG. Oral Oncol. (2020) 111:105012. doi: 10.1016/j.oraloncology.2020.105012, PMID: 32980659

[15] Wang M Sharma A Osazuwa-Peters N Simpson MC Schootman M Piccirillo JF . Risk of subsequent Malignant neoplasms after an index potentially-human papillomavirus (HPV)-associated cancers. Cancer Epidemiol. (2020) 64:101649. doi: 10.1016/j.canep.2019.101649, PMID: 31816512 PMC7415188

[16] Steeves RA Bataini JP . Neoplasms induced by megavoltage radiation in the head and neck region. Cancer. (1981) 47:1770–4. doi: 10.1002/1097-0142(19810401)47:7<1770::AID-CNCR2820470708>3.0.CO;2-7, PMID: 6784913

[17] Liu L Liu S Xia X Zheng L Zhang X Hu J . Association of radiotherapy with secondary pelvic cancers in male patients with rectal cancer. Int J Colorectal Dis. (2025) 40:65. doi: 10.1007/s00384-025-04840-x, PMID: 40075051 PMC11903567

[18] DiMarzio P Peila R Dowling O Timony DM Balgobind A Lee LN . Smoking and alcohol drinking effect on radiotherapy associated risk of second primary cancer and mortality among breast cancer patients. Cancer Epidemiol. (2018) 57:97–103. doi: 10.1016/j.canep.2018.10.002, PMID: 30359894

[19] Hawkins MM . Second primary tumors following radiotherapy for childhood cancer. Int J Radiat Oncol Biol Phys. (1990) 19:1297–301. doi: 10.1016/0360-3016(90)90248-I, PMID: 2254128

[20] Liang X Qin Y Li P Mo Y Chen D . Risk of second primary cancer in young breast cancer survivors: an important yet overlooked issue. Ther Adv Med Oncol. (2025) 17:17588359251321904. doi: 10.1177/17588359251321904, PMID: 40012707 PMC11863263

[21] Mazonakis M Lyraraki E Tolia M Damilakis J . Risk for second bladder and rectal Malignancies from cervical cancer irradiation. J Appl Clin Med Phys. (2021) 22:103–9. doi: 10.1002/acm2.13274, PMID: 34021692 PMC8292701

[22] Journy N Schonfeld SJ Hauptmann M Roberti S Howell RM Smith SA . Dose-volume effects of breast cancer radiation therapy on the risk of second oesophageal cancer. Radiother Oncol. (2020) 151:33–9. doi: 10.1016/j.radonc.2020.07.022, PMID: 32679305

